# Supplementary material for: Using mixture density networks to emulate a stochastic within-host model of Francisella tularensis infection
Source: PLoS Comput Biol. 2023 Dec 20;19(12):e1011266. doi: 10.1371/journal.pcbi.1011266 (PMC10766174; doi:10.1371/journal.pcbi.1011266)
Supplement: S1 Appendix — Applying the emulators to describe population-level infection dynamics. (PDF) [file pcbi.1011266.s001.pdf]

Supplementary material

Jonathan Carruthers, Thomas Finnie

Zonal-ventilation model

In Carruthers *et al.* (2018), a zonal-ventilation model is used to describe the concentration of *F. tularensis* bacteria in the air of a microbiology laboratory following its accidental release [1]. The doses of bacteria that individuals in the laboratory inhale are then provided as inputs to the within-host model to estimate the probability of disease. From this, it is then possible to determine the distribution of the number of individuals that would require treatment. Given that the within-host model parameter inference has now been repeated using records of human infection from Operation Whitecoat, here we also provide updated results from the zonal-ventilation model [2].

Consider a laboratory consisting of two rooms joined by a corridor, each of which are divided into two zones such that the air in each zone is assumed to be well-mixed (Fig 1, left). Let $C_j(t)$ represent the concentration of bacteria in the air in zone j at time t and $p_j(t)$ denote the cumulative amount of bacteria that an individual in zone j has inhaled at time t. By accounting for the volume of zone j, V_j, the flow of air in and out of zone j, β_{ij} and β_{ji}, and the extraction of air by ventilation systems, Q_j, the time evolution of C_j and p_j can be described using the system of ordinary equations (ODEs) in the right-hand panel of Fig 1. Here, n_j is the number of individuals in zone j and ρ is their respiratory rate.

To link the within-host model with the zonal-ventilation model, we previously showed that p_j reaches steady state on a much faster timescale than the infection dynamics. Therefore, the steady state value of p_j can be considered as the initial dose for individuals in zone j. Combining this with the improved estimates of the within-host model parameters allows us to apply the emulators developed in [2] to predict the probability

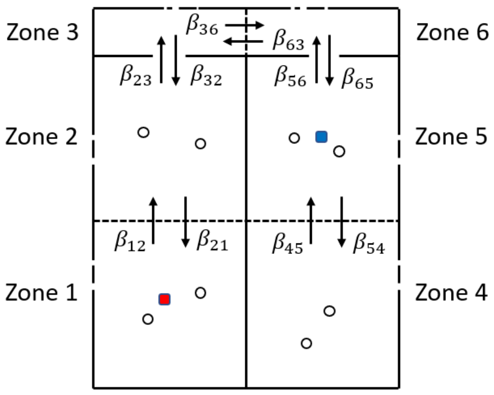

$$
\begin{aligned}
V_1\frac{dC_1}{dt} &= -\left(Q_1+\beta_{12}+\rho n_1\right)C_1+\beta_{21}C_2\\
V_2\frac{dC_2}{dt} &= -\left(Q_2+\beta_{21}+\beta_{23}+\rho n_2\right)C_2+\beta_{12}C_1+\beta_{32}C_3\\
V_3\frac{dC_3}{dt} &= -\left(Q_3+\beta_{32}+\beta_{36}\right)C_3+\beta_{23}C_2+\beta_{63}C_6\\
V_4\frac{dC_4}{dt} &= -\left(Q_4+\beta_{45}+\rho n_4\right)C_4+\beta_{54}C_5\\
V_5\frac{C_5}{dt} &= -\left(Q_5+\beta_{65}+\beta_{63}\right)C_6+\beta_{36}C_3+\beta_{56}C_5\\
\frac{dp_k}{dt} &= \rho C_k \qquad k=1,2,4,5
\end{aligned}
$$

Figure 1: Left: A diagram showing the setup of two rooms and a corridor within a laboratory, each of which is divided into two zones (dotted lines). Arrows indicate the flow of air between zones and dashed lines represent potential ventilation systems within each zone that extract air. Red and blue squares indicate the source of release for different scenarios, whilst circles are used to represent individuals. Right: The system of ODEs that dictates the airborne spread of bacteria between zones. The concentration of bacteria in zone j (C_j) increases due to airflow from neighbouring zones (β_{ij}) and decreases due to inhalation by individuals (ρ), air flowing to neighbouring zones (β_{ji}) and extraction (Q_j) [1].

Scenario	β_{ij} (m^3/min)	Q_i (m^3/min)	Source room	Steady state
A1	$\beta_{12} = \beta_{23} = \beta_{36} = \beta_{63} = \beta_{56} = \beta_{45}$<br>$= \beta_{21} = \beta_{32} = \beta_{65} = \beta_{54} = 9$	$Q_i = 3,\ i = 1, ..., 6$	1	$\boldsymbol{p} = (145, 82, 13, 17)$
A2	$\beta_{12} = \beta_{23} = \beta_{36} = \beta_{63} = \beta_{56} = \beta_{45}$<br>$= \beta_{21} = \beta_{32} = \beta_{65} = \beta_{54} = 9$	$Q_i = 3,\ i = 1, ..., 6$	5	$\boldsymbol{p} = (17, 23, 82, 110)$
C1	$\beta_{12} = \beta_{23} = \beta_{36} = \beta_{63} = \beta_{56} = \beta_{45} = 9$<br>$\beta_{21} = \beta_{32} = \beta_{65} = \beta_{54} = 18$	$Q_i = 9,\ i = 1, ..., 4$<br>$Q_i = 0,\ i = 5,\ 6$	1	$\boldsymbol{p} = (102, 46, 9, 9)$
C2	$\beta_{12} = \beta_{23} = \beta_{36} = \beta_{63} = \beta_{56} = \beta_{45} = 9$<br>$\beta_{21} = \beta_{32} = \beta_{65} = \beta_{54} = 18$	$Q_i = 9,\ i = 1, ..., 4$<br>$Q_i = 0,\ i = 5,\ 6$	5	$\boldsymbol{p} = (18, 18, 92, 92)$

Table 1: Airflow parameters for the four scenarios considered, along with steady state bacterial intake values that represent the initial dose for individuals in each zone, $\boldsymbol{p} = \lim_{t\to\infty}(p_1(t), p_2(t), p_4(t), p_5(t))$ [1]. In all scenarios we assume that $\rho = 0.01 m^3/\text{min}$, $V_i = 36 m^3$ for $i = 1, 2, 4, 5$ and $V_i = 12 m^3$ for $i = 3, 6$.

of disease and distribution of incubation periods for each individual. Let π_j denote the probability of disease for individuals in zone j, then $X_j \sim \text{Binomial}(n_j, \pi_j)$ is the number of individuals in zone j that develop disease and $Z = \sum_j X_j$ is the total number of cases across the laboratory. The distribution of Z is provided in the left-hand panel of Fig 2 for the four different scenarios detailed in Table 1. In each of these scenarios we assume that at the time of release there are two individuals in zones 1, 2, 4 and 5. In [2], we noted that the probability of disease calculated in [1] is an underestimate, which explains why the distribution of total cases has now shifted towards larger values. Despite this, we reach the same conclusions regarding the optimal ventilation regime, where the location of the source zone and the increased extraction of air explain why scenario C1 yields the fewest expected cases.

When left untreated, mortality rates of 30% have been reported from *F. tularensis* infections with the more severe type A strains, such as Schu S4. However, antibiotic treatment has reduced the fatality rate in the United States to less than 2% [3]. Since early treatment increases the chance of survival, the minimum incubation period can be used to understand the time-frame for effective intervention. Let $F_{X_j}(t)$ denote the cumulative density function (cdf) of the incubation period for individuals in zone j and $F_{\min}(t)$ be the

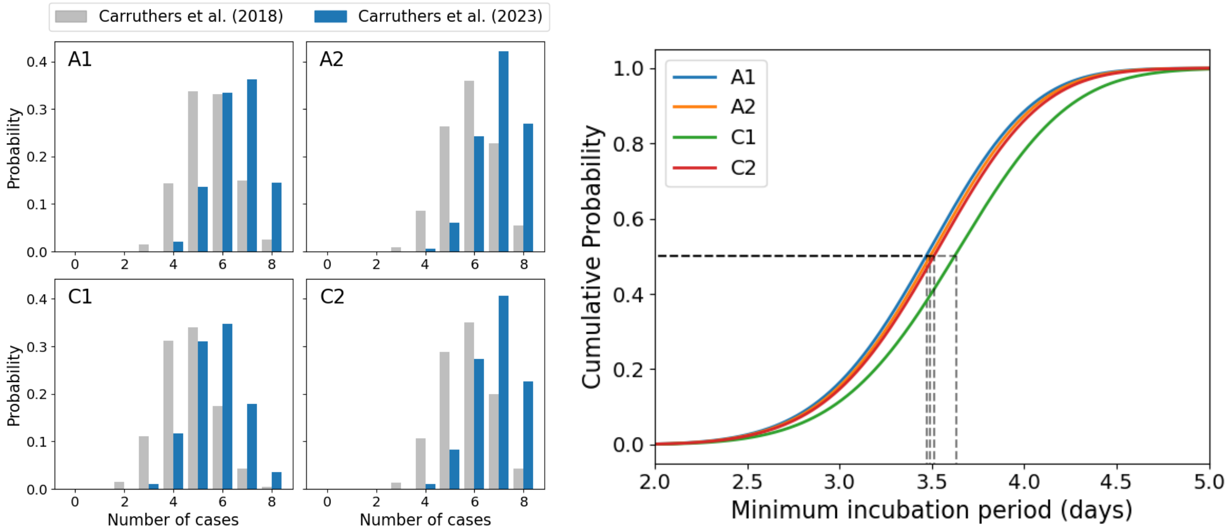

Figure 2: Left: The distribution of the number of individuals that develop disease within the laboratory for four different scenarios. The distributions are shown for the original parameterization of the within-host model (grey) and the updated parameterization inferred from records of human infection (blue). Right: The cumulative probability of the minimum incubation period for each of the four scenarios. Dashed lines indicate the median minimum incubation period.

cdf of the minimum incubation period. For the laboratory example, it can be shown that

$$F_{\min}(t) = \sum_{i=0}^{n_1} \sum_{j=0}^{n_2} \sum_{k=0}^{n_4} \sum_{l=0}^{n_5} \Pr(X_1 = i, X_2 = j, X_4 = k, X_5 = l) \left[1 - (1 - F_{X_1}(t))^i (1 - F_{X_2}(t))^j (1 - F_{X_4}(t))^k (1 - F_{X_5}(t))^l\right] .$$

The right-hand panel of Figure 2 shows the distribution of the minimum incubation period for each of the four scenarios considered in Table 1. The doses that individuals in different zones inhale do not vary greatly between scenarios, therefore only subtle differences in the minimum incubation period are observed. Note that without the mixture density network, equivalent results could only be obtained through time-consuming simulations of the within-host model.

Whilst the zonal-ventilation model described here simplifies the airflow dynamics by only partitioning each room into two zones, it provides a practical application of the *F. tularensis* within-host model that can be extended using more realistic models for the airborne spread of bacteria. Furthermore, the benefits of emulating the incubation period distribution using a mixture density network are again demonstrated through the calculation of the minimum symptom onset time.

References

[1] Carruthers J, López-García M, Gillard JJ, Laws TR, Lythe G, Molina-París C. A novel stochastic multi-scale model of Francisella tularensis infection to predict risk of infection in a laboratory. Frontiers in Microbiology. 2018; 9: 1165. https://doi.org/10.3389/fmicb.2018.01165

[2] Carruthers J, Finnie T. Using mixture density networks to emulate a stochastic within-host model of Francisella tularensis infection. https://doi.org/10.1371/journal.pcbi.1011266.

[3] Oyston PCF, Sjöstedt A, Titball RW. Tularaemia: bioterrorism defence renews interest in Francisella tularensis. Nature Reviews Microbiology. 2004; 2(12): 967–978. https://doi.org/10.1038/nrmicro1045
